# Macrophages modulate skeletal muscle wasting and recovery in acute lung injury in mice

**DOI:** 10.14814/phy2.70052

**Published:** 2024-09-26

**Authors:** Jennifer T. W. Krall, Lanazha Belfield, Claire Strysick, Chun Liu, Lina Purcell, Renee Stapleton, Michael Toth, Matthew Poynter, Xuewei Zhu, Kevin Gibbs, D. Clark Files

**Affiliations:** ^1^ Section on Pulmonary, Critical Care, Allergy, and Immunologic Disease, Department of Medicine Wake Forest University School of Medicine Winston‐Salem North Carolina USA; ^2^ Division of Pulmonary and Critical Care Medicine, Department of Medicine University of Vermont College of Medicine Burlington Vermont USA; ^3^ Section on Molecular Medicine, Department of Medicine Wake Forest University School of Medicine Winston‐Salem North Carolina USA

**Keywords:** acute lung injury, acute respiratory distress syndrome, intensive care unit acquired weakness, macrophage, skeletal muscle wasting

## Abstract

Skeletal muscle dysfunction in critical illnesses leaves survivors weak and functionally impaired. Macrophages infiltrate muscles; however, their functional role is unclear. We aim to examine muscle leukocyte composition and the effect of macrophages on muscle mass and function in the murine acute lung injury (ALI)‐associated skeletal muscle wasting model. We performed flow cytometry of hindlimb muscle to identify myeloid cells pre‐injury and time points up to 29 days after intratracheal lipopolysaccharide ALI. We evaluated muscle force and morphometrics after systemic and intramuscular clodronate‐induced macrophage depletions between peak lung injury and recovery (day 5–6) versus vehicle control. Our results show muscle leukocytes changed over ALI course with day 3 neutrophil infiltration (130.5 ± 95.6cells/mg control to 236.3 ± 70.6cells/mg day 3) and increased day 10 monocyte abundance (5.0 ± 3.4%CD45^+^CD11b^+^ day 3 to 14.0 ± 2.6%CD45^+^CD11b^+^ day 10, *p* = 0.005). Although macrophage count did not significantly change, pro‐inflammatory (27.0 ± 7.2% day 3 to 7.2 ± 3.8% day 10, *p* = 0.02) and anti‐inflammatory (30.5 ± 11.1% day 3 to 52.7 ± 9.7% day 10, *p* = 0.09) surface marker expression changed over the course of ALI. Macrophage depletion following peak lung injury increased muscle mass and force generation. These data suggest muscle macrophages beyond peak lung injury limit or delay muscle recovery. Targeting macrophages could augment muscle recovery following lung injury.

## 1 INTRODUCTION

Skeletal muscle dysfunction is a common complication of critical illnesses such as acute respiratory distress syndrome (ARDS) and leaves some survivors with prolonged muscle weakness (Herridge & Azoulay, 2023; Palakshappa et al., 2021). Over a third of survivors of ARDS and other critical illnesses experience impaired functional recovery through hospital discharge and as far out as 5 years (Dinglas et al., 2017; Fan et al., 2014; Herridge et al., 2011). While the body of research linking muscle wasting, ARDS, and other critical illnesses has grown over the past several years, mechanisms underlying muscle weakness associated with ARDS and other critical illnesses remain poorly understood (Files et al., 2012; Files, Sanchez, & Morris, 2015; Gandotra et al., 2019; Palakshappa et al., 2024; Puthucheary et al., 2013).

ARDS, an acute inflammatory lung injury caused by various clinical insults, results in profound respiratory failure and widespread, extrapulmonary, systemic inflammation (Matthay et al., 2019, 2024). Pro‐inflammatory cytokines present in plasma during ARDS are associated with acute indirect skeletal muscle injury (Janz & Ware, 2013). Additionally, pro‐inflammatory cytokines mediate activation of transcription factor nuclear factor kappa light chain enhancer of activated B cells (NF‐κB) and increase muscle protein degradation (Ochala et al., 2011; Paddon‐Jones et al., 2006; Puthucheary et al., 2013). Whereas the major cellular drivers of these processes in skeletal muscle are poorly understood, serial muscle biopsies performed in critically ill patients found macrophage infiltration into the muscle (Puthucheary et al., 2013). Whether these macrophages contribute to skeletal muscle injury or repair in critically ill patients is unclear. Pre‐clinical models of direct skeletal muscle injury and repair are influenced by infiltrating myeloid cells, although the role of these cells in a pre‐clinical model of ARDS is unclear (Arnold et al., 2007; Ferrara et al., 1985).

To study the role of muscle myeloid cells in the context of lung injury, we utilized the well‐validated murine model of intratracheal lipopolysaccharide (IT‐LPS) experimental acute lung injury (ALI) (D'Alessio et al., 2009). IT‐LPS results in robust and reproducible lung inflammation with subsequent induction of muscle wasting, reproducing key features of muscle wasting in patients with ARDS (Files et al., 2012; Files, Liu, et al., 2015). Using the ALI‐associated skeletal injury model, we sought to characterize the changes in skeletal muscle leukocyte composition over the course of injury and recovery and determine the functional role of macrophages in muscle wasting and recovery.

## 2 MATERIALS AND METHODS

### 2.1 Animals and acute lung injury model

The Wake Forest School of Medicine Animal Care and Use Committee approved all protocols and procedures performed in this study. Eight‐week‐old C57BL/6 mice (The Jackson Laboratory, Bar Harbor, ME) were housed in a pathogen‐free facility. Mice were housed on a 12/12‐h light/dark cycle with ad libitum access to standard chow (ProLab RMH 3000—5P00) and water. To induce lung injury, mice were anesthetized with an intraperitoneal (IP) injection of 150 mg/kg ketamine and 13.5 mg/kg acetylpromazine, and the trachea was exposed. *Escherichia coli* lipopolysaccharide (LPS) (O55:B5 L2880; Sigma‐Aldrich, St. Louis, MO) or sterile water (sham) at 3 μg/g mouse was then instilled intratracheally (IT) using a 20‐gauge catheter as previously described (Files et al., 2012). The wound was closed with glue. Recovery was monitored following anesthesia. Control mice were naïve animals that did not undergo IT LPS.

### 2.2 Bronchoalveolar lavage analysis

At specified times (control, sham, days 3, 10, and 25–29 after instillation of LPS), mice were anesthetized, bronchoalveolar lavage fluid (BALF) was collected, and the animals then euthanized by exsanguination. BALF cell counts were performed as previously described (D'Alessio et al., 2009).

### 2.3 Flow cytometry

Age matched mice that were housed together were randomly assigned to pre‐specified harvest times. At specified times (control, days 3, 10, and 25–29 after instillation of LPS, Figure 2a), mice were anesthetized, tibialis anterior muscles (TA) were collected, and the animals then euthanized by exsanguination. The TA was cut into small pieces with scissors and processed with a skeletal muscle dissociation kit according to manufacturer instructions (GentleMACS Dissociator, Miltenyi Biotec, Auburn, CA) to form a single‐cell suspension. After viability staining (LIVE/DEAD Fixable Yellow Dead Cell Stain Kit, Invitrogen, Cat#L34967) and FcBlock (Mouse BD Fc Block, BD Biosciences, Cat#553141), the cells were stained with fluorochrome‐conjugated surface antibodies: APC/Fire 750 anti‐mouse CD45 Antibody (BioLegend, Cat#103153), PE anti‐mouse/human CD11b Antibody (BioLegend, Cat#101207), PE/Cyanine7 anti‐mouse CD11c Antibody (BioLegend, Cat#117317), Brilliant Violet 421 anti‐mouse Ly‐6G Antibody (BioLegend, Cat#127627), APC anti‐mouse F4/80 Antibody (BioLegend, Cat#123115), Brilliant Violet 510 anti‐mouse Ly‐6C Antibody (BioLegend, Cat#128033), Brilliant Violet 785 anti‐mouse CD206 (MMR) Antibody (BioLegend, Cat#141729), CD163 Monoclonal Antibody (TNKUPJ), Super Bright 702 (Invitrogen, ThermoFisher, Cat#67–1631‐82), PE/Cyanine5 anti‐mouse I‐A/I‐E Antibody (BioLegend, Cat#107611). After staining, CountBright Absolute Counting Beads were added according to manufacturer instructions (Invitrogen, Cat#C36950) and the cells were resuspended in 250 μL FACS buffer (0.5% BSA in PBS) to bring the total volume of the flow tube to 300 μL. Flow cytometry analysis was performed on BD LSRFortessa X‐20 Analyzer and analyzed using FlowJo software (BD Biosciences). The live‐dead threshold was determined by mixing heat‐killed PBMC 1:1 with live PBMC. After gating for single cells, forward/side scatter, and live cells, macrophages were defined as CD45^+^CD11b^+^CD11c^−^Ly6G^−^F4/80^+^SSC^lo^Ly6C^lo^. Monocytes were defined as CD45^+^CD11b^+^CD11c^−^Ly6G^−^F4/80^+^SSC^lo^Ly6C^hi^. Neutrophils were defined as CD45^+^CD11b^+^CD11c^−^F4/80^−^Ly6G^+^. Dendritic cells were defined as CD45^+^CD11b^+^CD11c^+^. Macrophage and monocyte populations were defined using a combination of F4/80^+^ and gating previously described by Rose et al. (2012) Total intramuscular myeloid cells were quantified by CD45^+^CD11b^+^ (Rose et al., 2012). This was performed on control, day 3, 10, and 25–29 after ALI. Anti‐inflammatory macrophages were profiled as CD206^+^CD163^+^ (Ross et al., 2021; Tidball & Villalta, 2010; Viola et al., 2019). Pro‐inflammatory macrophages were profiled as MHC II^+^CD206^−^ and MHC II^+^CD163^−^ (Ross et al., 2021; Tidball & Villalta, 2010; Viola et al., 2019). Macrophage profiling was performed at control, day 3, and 10 after ALI. The gates for CD11c, F4/80, MHC II, CD163, and CD206 were determined by comparing cells stained with the antibodies to fluorescence minus one (FMO) control cells in which the antibody was omitted from the antibody cocktail (Figure S1d). Flow cytometry experiments were performed with control mice matched with the ALI time points. Male and female mice were included in this experiment and compared to ensure there were no significant sex‐based differences. A representative gating strategy is presented in Figure S1b,c.

### 2.4 Macrophage depletion

Liposomes (Liposoma, Amsterdam, Netherlands) were stored at 4°C. Prior to injection, the clodronate‐containing liposomes (5 mg/mL) and PBS‐containing liposomes (vehicle control) were removed from the refrigerator and allowed to acclimate to room temperature (18°C). The tubes containing liposomes were inverted 8–10 times to ensure even distribution. For intramuscular depletion, liposomes were dosed at 20 μL of solution per leg (100 μg clodronate). The mouse fur was removed from bilateral lower limbs overlying the tibialis anterior (TA) muscle compartment by applying depilatory cream (Nair). Skin was cleaned with 70% ethanol and the dosing solution injected in four aliquots along the body of the TA muscle compartment, as previously described (Krall et al., 2022). The contralateral muscle was injected with equivalent amount of vehicle control. This has been reported to decrease muscle macrophages by about 60 to 80% using this protocol (Gong et al., 2016; Krall et al., 2022). Intramuscular macrophage depletion was performed at 6 days and 8 days following ALI. Mice were euthanized at 10 days following ALI. For systemic depletion, liposomes were dosed at 100 μL of solution per 10 grams of animal weight per manufacturer protocol. Abdominal skin was cleaned with 70% ethanol, and the dosing solution was injected intraperitoneally. Systemic macrophage depletion was performed at 5 days following ALI (DiPasquale et al., 2007; Liu et al., 2019; Tyner et al., 2005). Mice were euthanized at 8 days following ALI. Male mice were used in these experiments.

### 2.5 Muscle characterization and morphometric analysis

Following euthanasia, the tibialis anterior muscle was isolated. The wet weight of the tibialis anterior (TA) muscle was assessed to evaluate loss of muscle mass. For morphometric analyses, the tibialis anterior muscle was affixed at resting length and frozen in isopentane equilibrated in liquid nitrogen and mounted for cyosectioning in OCT. Sections of 10 μm thickness were obtained from the mid‐belly of the muscle and incubated with an antibody to laminin‐γ1 (antibody clone A5, Millipore Sigma, Cat#MAB1914P) followed by an Alexa Fluor 488‐conjugated secondary antibody (Invitrogen, ThermoFisher, Cat#A‐11006). Five non‐overlapping images of the muscle cross‐section were taken at 20X magnification with a reference bar of 200 μm and minimal cross‐sectional area (CSA, cross section of muscle perpendicular to its fibers) were determined using Image J software by overlying ATPase and Laminin images. Scale set based on distance in pixels, known distance, pixel aspect ratio. The data were then expressed as a distribution of the percentage of the total number of myofibers analyzed or as mean fiber CSA.

To process samples for immunofluorescence of myosin heavy chain isoforms (Bonilla et al., 2020), sections were rinsed in PBST and blocked with 10% goat serum in PBS for 1 h. The mouse primary antibodies used for MHC isoforms, developed by Dr. Stefano Schiaffino (Schiaffino et al., 1989) and purchased through the University of Iowa Developmental Studies Hybridoma Bank, include BA‐F8 (MHC‐I, 1:50), SC‐71 (MHC‐IIa, 1:500), and BF‐F3, (MHC‐IIb, 1:100). To mark the extracellular space, laminin‐γ1 (1:100, Millipore Sigma) staining was used. Primary antibodies were incubated at room temperature for 2 h with 10% goat serum in PBS. Sections were washed three times in PBST for 5 min and incubated with secondary goat anti‐mouse antibodies for MHC: AlexaFluor 350 IgG2b (1:500, Cat#A‐21140), AlexaFluor 488 IgG (1:500, Cat#A‐21121), and AlexaFluor 555 IgM (1:500, Cat#A‐21426). Goat anti‐rat IgG Alexa Fluor 680 (1:500, Cat#A‐21096) secondary antibody was used for laminin. Secondary antibodies were obtained from Invitrogen (ThermoFisher Scientific, Carlsbad, CA). Slides were washed in PBS, dried, and mounted using the Dako fluorescence mounting medium (Dako North America, Carpinteria, CA). Imaging was performed by the Wake Forest School of Medicine Cellular Imaging Shared Resource. Analysis with a four‐channel overlay was performed to determine minimal cross‐sectional area using Image J software. Fiber types were identified as types IIX (black), IIA (green), IIB (red), IIX/A (intermediate green), and IIX/B (intermediate red) (Figure 4f). The data were then expressed as mean fiber CSA.

### 2.6 Ex vivo isolated skeletal muscle force and Fatiguability measurement

We measured muscle force production by determining the ex vivo contractility of the isolated soleus muscle. Following anesthesia, soleus muscle was isolated, and the proximal and distal ligaments were cut. The animals were euthanized by exsanguination. The muscle was secured with aluminum foil at the proximal and distal tendons and placed into a petri dish containing Kreb's buffer. The soleus muscle was mounted in the bath chamber containing 25 mL of running buffer (composition of 1 liter (L): 2 molar (M) NaCl 0.0675 L, 1 M KCl 0.0059 L, 0.2 M MgSO_4_ 0.0075 L, 0.2 M CaCl_2_ 0.006 L, 0.0552 g/L NaH_2_PO_4_, 2.016 g/L NaHCO_3_, 0.992gr/L glucose), continuously gassed with a mixture of 95% O_2_ and 5% CO_2_ maintained at room temperature. The proximal tendon was fixed to a force transducer (Aurora Scientific, Aurora, Ontario, Canada), and the distal tendon was fixed to a hook at the opposite side of the chamber. After 5–10 min of equilibration, the soleus was stretched to its optimal length, which is the length producing the maximal signal contraction (twitch). Tetanic contractions were obtained by applying pulses lasting 3–4 s at increasing frequencies (20, 40, 60, 80, 100, 125, 150, 200 Hz) every 1 min. The raw force generated is the absolute force. The data generated was represented in force‐frequency curves and tetanic force (at 100 Hz). The specific force was calculated by dividing the absolute force by the calculated muscle cross‐sectional area as previously described. (Brooks & Faulkner, 1988) Muscle fatigue was evaluated by applying a sequence of pulses at 150 Hz for 400 ms with an interval of 1 ms. Fatigue was calculated by determining the percent decline from max (peak) force (100%) for each subsequent force (referenced to the first force generated).

### 2.7 Statistical analysis

Data are expressed as the mean ± standard deviation (SD). A nonparametric t‐test (Mann–Whitney test) was performed to compare two groups at a single time point. A nonparametric paired t‐test (Wilcoxon) was performed to compare two groups at a single time point with paired data (i.e. within the mouse contralateral leg). A one‐way ANOVA (Kruskal‐Wallis test) was performed to analyze a single group at multiple time points. Post‐hoc comparisons between time points were performed using Dunn's multiple comparisons test. For multiple comparisons and force‐frequency curves, a two‐way ANOVA (mixed‐effects analysis) was performed. Differences were considered significant at *p* less than 0.05. Analyses were performed with GraphPad Prism 10. For comparison of the distribution of fibers between two groups, a Poisson regression model was fitted in R (version 4.1.1 *stats* package) to predict the count of fibers with a size greater than 600 μm^2^, including the individual animal as a covariate and total number of fibers counted as an offset.

## 3 RESULTS

### 3.1 Intratracheal lipopolysaccharide‐induced acute lung injury causes skeletal muscle wasting and reduces skeletal muscle function

**FIGURE 1:**
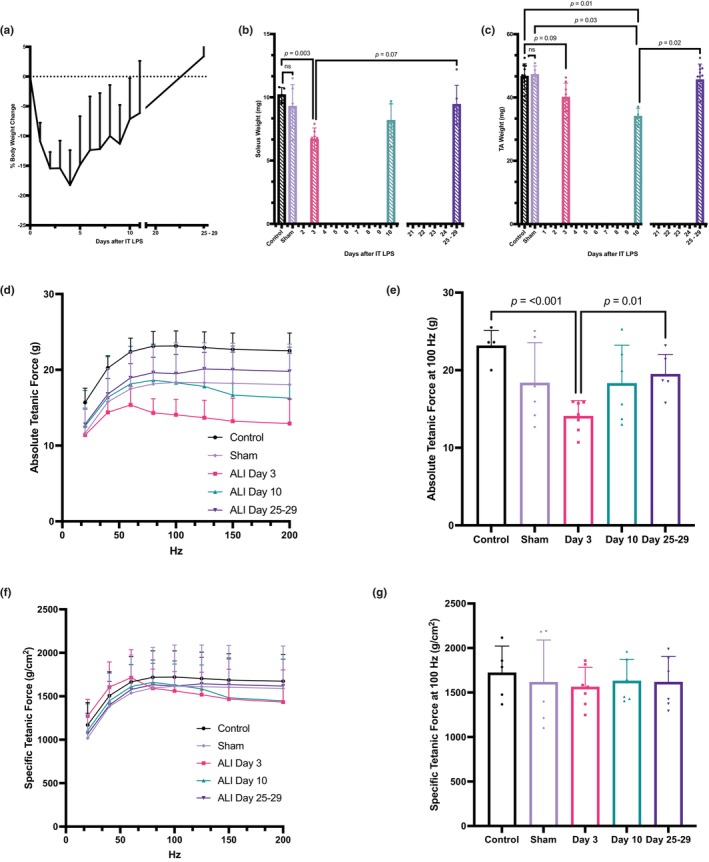
Effect of intratracheal lipopolysaccharide‐induced (IT LPS) acute lung injury (ALI) on skeletal muscle wasting and function over the course of acute lung injury and recovery. (a) Percent change in body weight from prior to IT LPS by repeated measures of individual mice prior to harvest (*n* = 35). (b) Soleus wet weight at harvest without ALI (control, *n* = 5), sham (*n* = 6), and at time points after ALI (ALI day 3 *n* = 7, ALI day 10 *n* = 6, ALI day 25–29 *n* = 6). (c) Tibialis Anterior (TA) wet weight without ALI (control, *n* = 11), sham (*n* = 5), and at time points after ALI (ALI day 3 *n* = 7, ALI day 10 *n* = 4, ALI day 25–29 *n* = 12). (d) Ex vivo absolute tetanic force measured over a range of stimulation frequencies of the soleus muscle without ALI (control, *n* = 5), sham (*n* = 6), and at time points after ALI (ALI day 3 *n* = 7, ALI day 10 *n* = 6, ALI day 25–29 *n* = 6). (e) Absolute tetanic force at the stimulation frequency of 100 Hz. (f) Ex vivo specific tetanic force (absolute force normalized to muscle cross‐sectional area) measured over a range of stimulation frequencies of the soleus muscle without ALI (control, *n* = 5), sham (*n* = 6), and at time points after ALI (ALI day 3 *n* = 7, ALI day 10 *n* = 6, ALI day 25–29 *n* = 6). (g) Specific tetanic force at the stimulation frequency of 100 Hz. (a–g) Data points are shown as mean ± SD. One‐way ANOVA (b, c, e, g) or two‐way ANOVA (d, f) was performed. Between time point post‐hoc comparisons were performed using Dunn's multiple comparisons test (b, c, e, g).

Intratracheal LPS induces a lung injury pattern characterized by peak cellular infiltrates on day 3 and by lung injury resolution by day 10 (Files et al., 2012). Our study recapitulated these findings (Figure S1a). Our lab has previously demonstrated that tibialis anterior (TA) muscle function by in vivo force measurement does not recover to baseline by post‐injury day 10 (Files et al., 2012). In the current study, we examined the timepoint of 25–29 days after ALI, when alveolar cell counts and composition were found to be similar to pre‐injury (6.2 × 10^4^ ± 4.1 × 10^4^ vs. 2.4 × 10^4^ ± 9.9 × 10^3^, Figure S1a). Intratracheal LPS induced an approximately 20% decrease in mouse total body weight in the first 4 days (Figure 1a), followed by an increase of body weight that was still below pre‐injury body weight at day 10. By day 25, body weight had recovered to above the pre‐injury body weight.

Intratracheal LPS also significantly induced the weight loss of soleus (Figure 1b) and TA (Figure 1c) muscle, which recovered to levels similar to pre‐injury weight by day 25 of ALI. We observed a 39% reduction in absolute tetanic force between control and ALI day 3 (Figure 1d,e) at tetanic force (100 Hz), and an increase in absolute force by day 25–29 (14.1 ± 2.0 g ALI day 3 vs 19.5 ± 2.6 g, *p* = 0.01). At day 25–29, the mean absolute force of soleus muscle (19.5 ± 2.6 g) was still lower than control (23.1 ± 2.0 g), although there is no significant difference between groups. No significant difference was observed in specific force (absolute force normalized to muscle cross‐sectional area) over the time course of ALI, suggesting decreased muscle force generation is primarily caused by muscle atrophy (Figure 1f,g).

### 3.2 Intramuscular leukocyte abundance and composition changes over the time course of ALI

**FIGURE 2:**
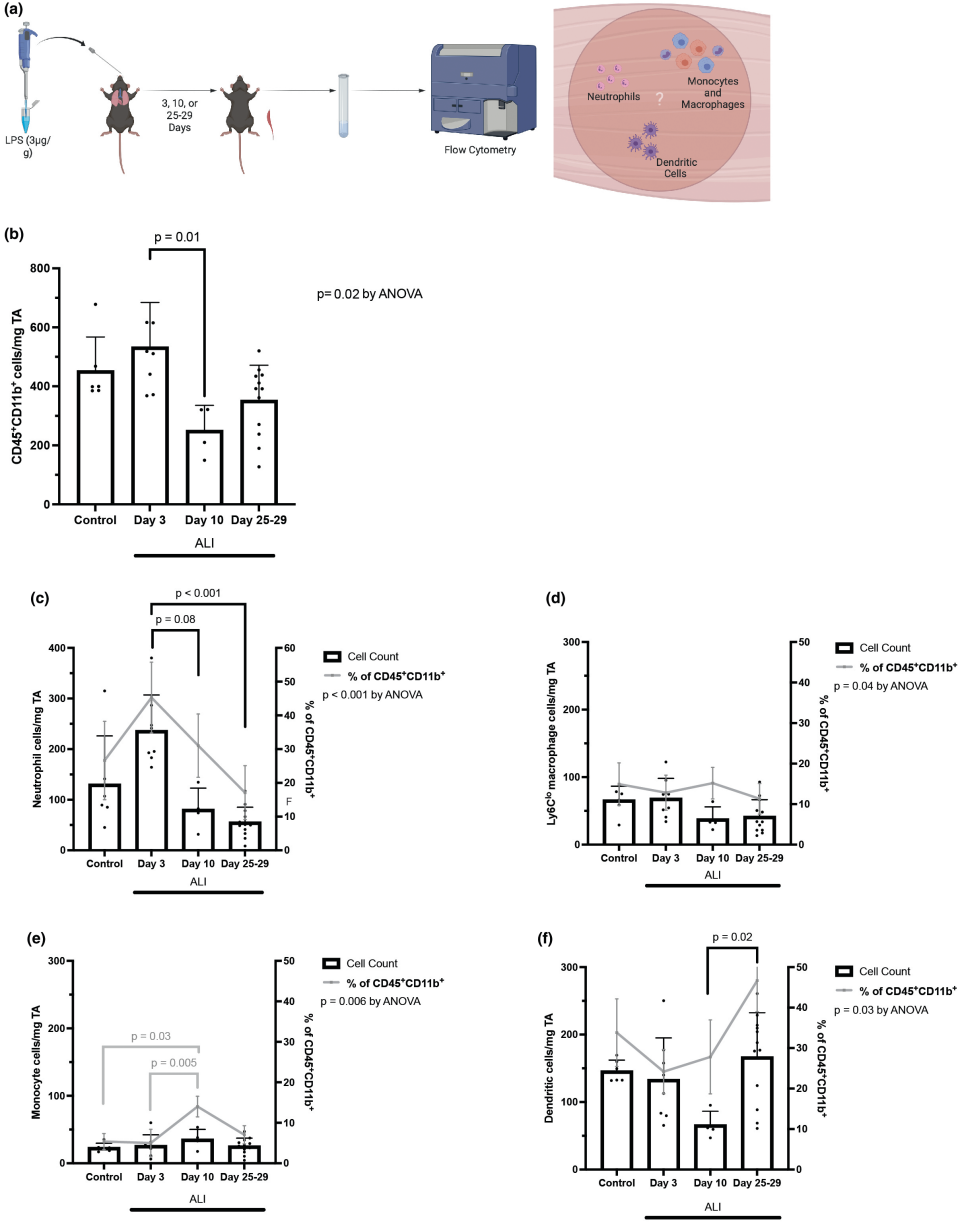
Leukocytes in tibialis anterior muscles were analyzed prior to ALI (control) and at time points over the course of acute lung injury and recovery. (a) Mice received intratracheal LPS and muscle analyzed by flow cytometry at days 3, 10, and 25–28. Abundance of (b) CD45^+^CD11b^+^ leukocyte, (c) neutrophil, (d) macrophage, (e) monocyte, and (f) dendritic cell counts were measured by flow cytometry and are shown normalized to the weight of the TA muscle. Muscles were analyzed from control mice (no ALI, *n* = 6), days 3 (*n* = 8), 10 (*n* = 4), and 25–29 (*n* = 12) of ALI. Data points are shown as mean ± SD. One‐way ANOVA was performed. Between time point post‐hoc comparisons were performed using Dunn's multiple comparisons test. Figure 2a created with BioRender.com.

ALI alters the composition and abundance of skeletal muscle myeloid cells. In our study, CD45^+^CD11b^+^ cells in the TA muscle tissues displayed a dynamic change in cell counts through day 25, with a non‐significant increase at day 3 (452.6 ± 114.6 cells/mg TA in control vs. 533.2 ± 151.3 cells/mg TA in day 3) and a significant decrease to 250 ± 85.2 cells/mg TA at day 10 (*p* = 0.011, Figure 2b). Specifically, neutrophil number increased from 130.5 ± 95.6 cells/mg TA in control to 236.3 ± 70.6 cells/mg TA at day 3 of ALI before returning to baseline at day 10 of ALI (Figure 2c). The changes in the absolute number of Ly6C^lo^ macrophages, which were generally low, in TA muscle, were less dynamic compared to neutrophils throughout the time course (Figure 2d). Interestingly, we observed a 2‐fold increase in monocytes from day 3 (5.0 ± 3.4% of CD45^+^CD11b^+^) to day 10 (14.0 ± 2.6% of CD45^+^CD11b^+^, *p* = 0.005) (Figure 2e). In contrast, dendritic cells decreased from day 3 (133.3 ± 61.7 cells/ mg TA) to day 10 (65.9 ± 20.6 cells/mg TA) and then increased between day 10 to day 25–29 (166.7 ± 65.7 cells/ mg TA, *p* = 0.02) (Figure 2f). Survival rate for this experiment was 89%. In summary, our results suggest a dynamic nature of intramuscular leukocyte abundance and composition over the time course of ALI.

### 3.3 Macrophage polarization changes dynamically over the time course of ALI

**FIGURE 3:**
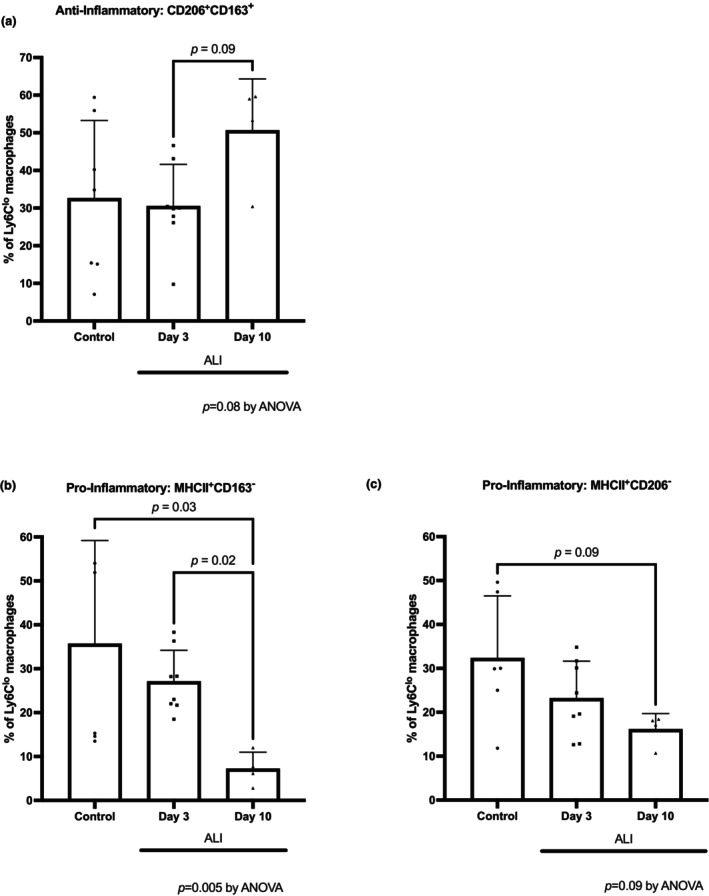
M1‐like pro‐inflammatory and M2‐like anti‐inflammatory surface markers were measured in skeletal muscle macrophages over the course of acute lung injury and recovery. Muscles were analyzed from control mice (no ALI, *n* = 6), days 3 (*n* = 8), 10 (*n* = 4), and 25–29 (*n* = 12) of ALI. Percent of total macrophages with CD163 and CD206 expression of M2‐like polarization (a, CD206^+^CD163^+^) and MHCII expression of M1‐like polarization (b, MHCII^+^CD163^−^ and c, MHCII^+^CD206^−^). Data points are shown as mean ± SD. One‐way ANOVA was performed. Between time point post‐hoc comparisons were performed using Dunn's multiple comparisons test.

Macrophages are functionally heterogeneous and can be polarized towards pro‐inflammatory M1‐like and anti‐inflammatory M2‐like subsets (Ross et al., 2021; Tidball & Villalta, 2010; Viola et al., 2019), which can be differentiated by their surface marker expression. For example, CD163 and CD206 are typical markers of M2‐like polarization, and MHCII is a marker of M1‐like polarization (Ross et al., 2021; Tidball & Villalta, 2010; Viola et al., 2019). Although Ly6^lo^ macrophages showed no significant difference over the time course of ALI (Figure 2d) in our study, the percentage of the anti‐inflammatory M2‐like macrophages (CD206^+^CD163^+^) showed a trend towards increase in the later times of ALI, from 30.5 ± 11.1% on day 3 of ALI to 52.7 ± 9.7% on day 10 of ALI (*p* = 0.09, Figure 3a). Consistent with this result, the percentage of the pro‐inflammatory M1‐like macrophages (MHCII^+^CD163^−^) showed a significant decrease from 27.0 ± 7.2% at day 3 to 7.2 ± 3.8% at day 10 of ALI (*p* = 0.02, Figure 3b,c). Because macrophage polarization displays the most dynamic changes during this recovery phase of ALI between day 3 and day 10, we chose this phase to target macrophages in the following experiments.

### 3.4 Macrophage depletion following lung injury increases muscle fiber size and force generation

**FIGURE 4:**
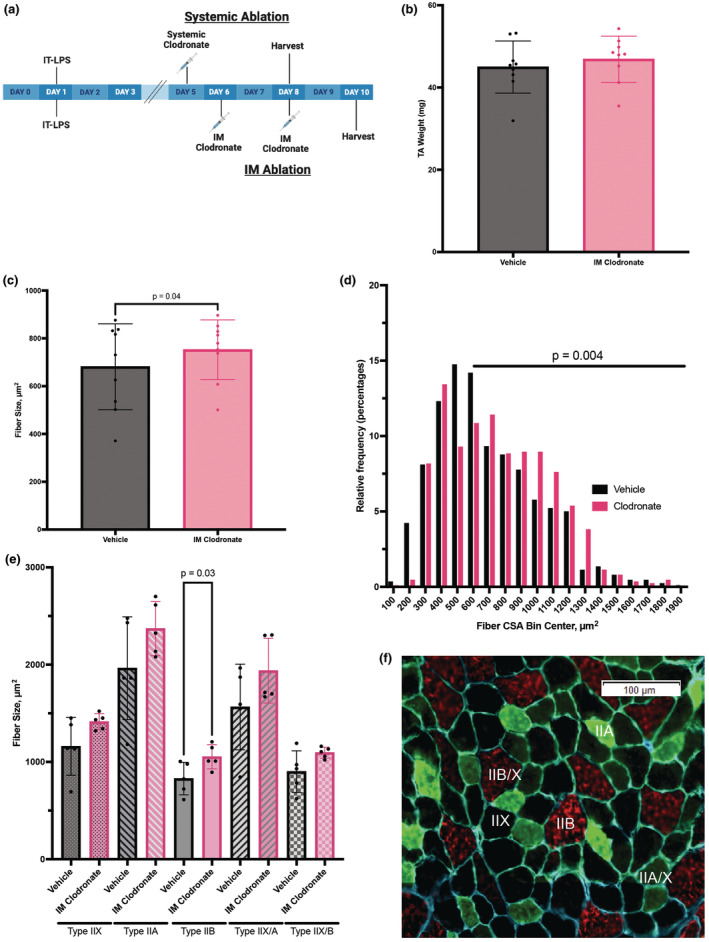
Intramuscular (IM) macrophage depletion with clodronate liposomes on was performed starting on ALI day 6 with analysis on ALI day 10 (a) (*n* = 9). Contralateral TA muscle was treated with vehicle control liposomes. (b) Tibialis Anterior (TA) wet weight (*n* = 9). For morphometric analyses, the tibialis anterior muscle cryosections were stained for laminin‐γ1 and cross‐sectional area (CSA) was determined. The data were then expressed as (c) mean fiber CSA or as (d) distribution of CSA of the total number of myofibers analyzed. (e) The tibialis anterior muscle cryosections were also stained for MHC isoforms and expressed as mean fiber CSA for each subtype: Types IIX, IIA, IIB, IIX/A, and IIX/B. (f) Representative tibialis anterior muscle MHC stain with types IIX (black), IIA (green), IIB (red), IIX/A (intermediate green), and IIX/B (intermediate red). Values are expressed as mean ± SD. Wilcoxon signed‐rank test (paired nonparametric *t* test) was performed (b, c). For comparison of the distribution of fibers between two groups, a Poisson regression model was fitted in R (version 4.1.1 *stats* package) to predict the count of fibers with a size greater than 600 μm^2^, including the individual animal as a covariate and total number of fibers counted as an offset (d). Mann Whitney test (unpaired nonparametric *t* test) was performed between treatments for each fiber type (e). Figure 4a created with BioRender.com.

To study the role of macrophages in muscle wasting and recovery, we depleted macrophages on days 5–6 post intratracheal LPS administration (Figure 4a), a period showing the most dynamic difference in M1/M2 macrophage polarization (Figure 3). Another reason for choosing this period is that we tried to avoid off‐target depletion of lung macrophages, which are key mediators of lung injury resolution (D'Alessio et al., 2016). Further, macrophages play a role in healthy muscle remodeling; thus, it was important for skeletal muscle regeneration not to deplete macrophages too quickly following injury. To do this, we injected intramuscular clodronate into the tibialis anterior muscle of the mice, and vehicle control was applied to the contralateral muscle on days 6 and 8 of ALI. Survival rate for this experiment was 96%. We observed a significant decrease in the mean fluorescent intensity of F4/80 in the CD11b^+^ cell population, indicating muscle macrophage depletion (Figure S2 a,b). There was no significant difference in wet weight of intramuscular vehicle or clodronate treated TA muscle (Figure 4b). Intramuscular macrophage depletion in the tibialis anterior increased the average fiber size cross‐sectional area from 681.0 ± 179.7 μm^2^ to 752.2 ± 125.1 μm^2^ (*p* = 0.04, Figure 4c). It also induced a rightward shift in fiber size distribution (Figure 4d), indicating in a greater distribution in fibers with larger cross‐sectional area. We fitted a Poisson model to predict the count of fibers with a size greater than 600 μm^2^ with the experimental condition and included individual animals as a covariate and a total number of fibers counted as an offset. Within this model, the effect of clodronate was statistically significant and positive (beta = 0.18, 95% CI [0.06, 0.30], *p* = 0.004, Std. beta = −0.35), further supporting a greater distribution in larger fibers with macrophage depletion. Furthermore, the mean fiber cross‐sectional areas were increased in all fiber subtypes, with a significant increase in type IIB in muscle macrophage‐depleted mice (Figure 4e,f).

**FIGURE 5:**
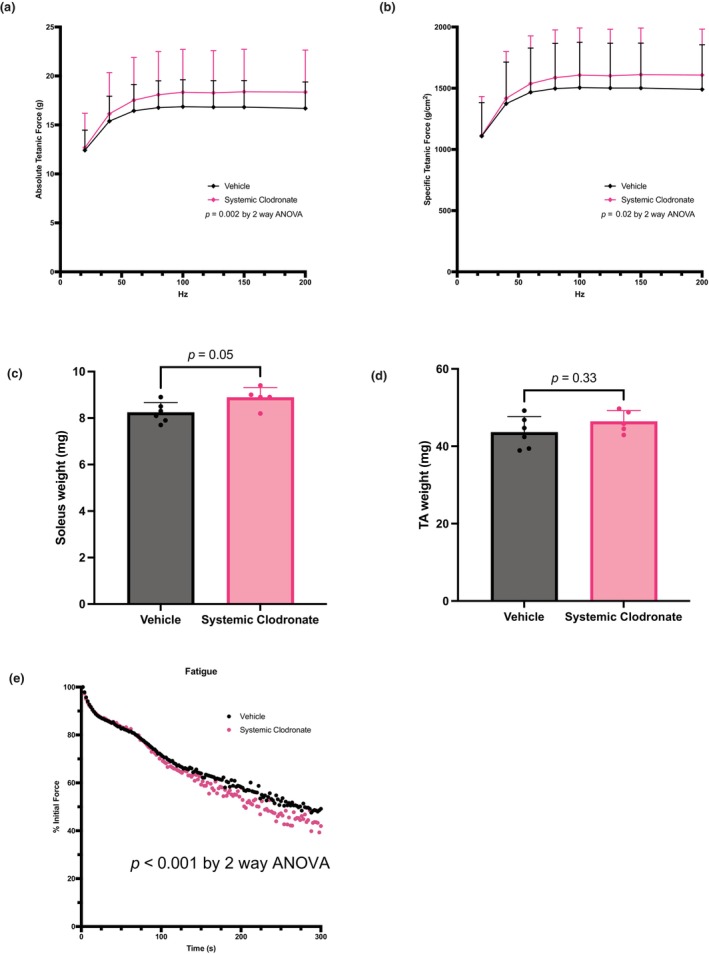
Systemic macrophage depletion with clodronate liposomes was performed starting on ALI day 5 with analysis on ALI day 8 (Figure 4a). (a) Ex vivo absolute tetanic force measured over a range of stimulation frequencies of the soleus muscle in mice treated with systemic clodronate (*n* = 5) and vehicle control (*n* = 6). (b) Ex vivo specific tetanic force (absolute force normalized to muscle cross‐sectional area) measured over a range of stimulation frequencies of the soleus muscle in mice treated with systemic clodronate (*n* = 5) and vehicle control (*n* = 6). (c) The wet weight of the soleus muscle was measured in muscle treated with systemic clodronate and vehicle control. (d) The wet weight of the TA muscle was measured in muscle treated with systemic clodronate and vehicle control. (e) Muscle fatigue was measured as a percentage of the initial peak force for each subsequent force. Values are expressed as mean ± SD. Wilcoxon test (c) or two‐way ANOVA (a, b, d) was performed.

In a separate experiment, we systemically administrated clodronate to the animals on day 5 of ALI and evaluated the muscle force generation on day 8. Survival rate for this experiment was 92%. Systemic macrophage depletion significantly increased the absolute and specific force‐frequency curve (Figure 5a,b), with an 8.7% increase in absolute force (Figure 5a) and a 6.8% increase in specific force (Figure 5b) at 100 Hz. Systemic clodronate‐induced macrophage depletion led to a significant increase in soleus wet weight by 7.9% (Figure 5c) and consistently a significant decrease in the time to fatigue curve (Figure 5d). Notably, Systemic clodronate administration did not affect lung injury or change total cell count or monocyte/macrophage percentages in the bronchoalveolar lavage (Figure S2 c,d). Collectively, our results suggest that local or systemic macrophage depletion following lung injury increases muscle fiber size and force generation.

Our previous study has shown in early ALI, muscle atrophy is driven by E3 ubiquitin‐ligase MuRF1 (Files et al., 2012). There was no significant difference in MuRF1 transcription between vehicle treated and clodronate treated muscles in either systemic or local depletion (Figure S3a,b). This suggests that the effect we are seeing from macrophage depletion appears to be independent of this pathway.

## 4 DISCUSSION

Macrophages play a crucial role in skeletal muscle injury and recovery in various models, and the loss of macrophage function can affect the immune response, injury, and recovery (Arnold et al., 2007; Ferrara et al., 1985; Tidball & Villalta, 2010). Our current study demonstrated the dynamic changes in leukocyte composition and abundance in skeletal muscle in a murine model of acute lung injury (ALI) with associated skeletal muscle wasting. Furthermore, reduction of macrophages during the recovery period of ALI helped mitigate skeletal muscle atrophy and weakness.

More than half of patients with critical illness develop muscle weakness (Palakshappa et al., 2021). Within the first week of critical illness, patients lose approximately 2% of their muscle cross‐sectional area, which in the second week increases to about 30% loss (Fazzini et al., 2023) The murine model of ALI‐associated muscle wasting robustly displays many key features of human critical illness‐associated muscle wasting, including myosin loss and type 2 myofiber atrophy (Files et al., 2012). The previous work by our group and the current study clearly show that skeletal muscle mass significantly decreases shortly after IT‐LPS, and recovery lags behind recovery from lung injury (Files et al., 2012). There is a concomitant reduction in absolute tetanic force produced by the isolated skeletal muscle, with force recovery occurring in the late time course of ALI. There was no significant change in specific force at any point examined post‐LPS, suggesting the decreased force generation is driven by muscle wasting.

Whereas serial muscle biopsies from patients with critical illness‐associated muscle weakness demonstrate that some patients have intramuscular macrophages, the functional role of these cells in this setting is unknown (Puthucheary et al., 2013). Our data from the murine model of ALI‐associated muscle wasting show that muscle leukocyte composition, specifically neutrophils, macrophages, monocytes, and dendritic cells, dynamically change in composition and abundance during this time course. Macrophage expression of polarization surface markers also change over this time course, with a decrease in pro‐inflammatory polarization and an increase in anti‐inflammatory polarization after peak lung injury.

In direct injury models, muscle injury repair has been shown to be orchestrated by an inflammatory response often initiated by neutrophil infiltration followed by macrophage infiltration (Tidball & Villalta, 2010). Macrophages undergo polarization to perform diverse functions in response to the surrounding microenvironment, classically categorized broadly into pro‐inflammatory or anti‐inflammatory functional phenotypes (Ross et al., 2021; Viola et al., 2019). During the repair process, macrophages phagocytose debris and produce cytokines and growth factors that support skeletal muscle regeneration. Macrophages can also contribute to muscle pathology and chronic injury (Tidball & Villalta, 2010). In our model, depletion of macrophages following peak lung injury improved muscle mass and increased muscle force generation. These data suggest that muscle macrophages beyond peak lung injury limit muscle recovery.

Our findings contribute to the growing body of literature on the complex mechanisms of skeletal muscle wasting and recovery in ARDS and other critical illnesses. Past studies have shown muscle atrophy in ARDS is driven by inflammation and disuse, and histologically show type 2 myofiber atrophy, myosin loss, or immune cell infiltrates (Files et al., 2012; Puthucheary et al., 2013). In a murine influenza pneumonia model, skeletal muscle macrophages contribute to the failure of skeletal muscle recovery in aged mice after influenza A infection (Runyan et al., 2020). These studies and ours highlight the potential critical role of leukocytes, specifically macrophages, in the injury and repair of skeletal muscle in ARDS and other critical illnesses.

We acknowledge the limitations of this study. We did not analyze the ontogeny of the macrophages in this study, limiting our ability to distinguish the relative contributions of monocyte‐derived macrophages and tissue‐resident macrophages. Our flow cytometry gating strategy has limitations in distinguishing between circulating monocytes and macrophages and resident macrophages as well as pro‐ and anti‐inflammatory phenotypes. Ly6C^high^ has been used in some studies as a marker for pro‐inflammatory phenotype so the pro‐inflammatory gating performed may underestimate the population. Further, we do not distinguish transitional monocytes or immature macrophages. Additionally, whereas clodronate liposomes have been used in many studies for the depletion of macrophages, they have recently been recognized to also affect neutrophils (Culemann et al., 2023). The timing of the clodronate after peak lung injury and utilizing both systemic and intramuscular clodronate may reduce some of the off‐target effects on neutrophils. Only male mice were used in the clodronate experiments which can be a potential limitation. We used mean fluorescence intensity (MFI) of F4/80 in the CD11b^+^ cell population, indicating muscle macrophage depletion (Figure S2 a,b). We have shown in a previous study muscle macrophage depletion in this technique measured by another method (Krall et al., 2022). Data is not available in the present study and timepoint and is a limitation of this study. The study uses muscles with differing fiber type compositions (soleus versus tibalis anterior) due to the techniques employed and this is may also be a limitation of this study as well. Future directions of study would include functional analysis of skeletal muscle macrophages, genetic lineage tracing, and transcriptomic analysis. There was no significant difference in muscle MuRF1 transcription, suggesting the effects seen by macrophage depletion may not be driven by MuRF1. This warrants further exploration. Another future direction given the dynamic neutrophil response, would be further experiments to study the role of neutrophils. Further analysis of skeletal muscle response would include further assessment of muscle atrophy such as analysis of gene and protein expressions of atrogenes.

In summary, our data show the murine ALI‐associated muscle wasting model has dynamic changes in leukocyte composition associated with skeletal muscle wasting and dysfunction. Macrophage polarization changes during this time course and appears to limit muscle recovery beyond peak lung injury. Targeting muscle macrophage number or phenotype could augment muscle recovery following lung injury.

## CONFLICT OF INTEREST STATEMENT

The authors declare no conflicts of interest.

## ETHICS STATEMENT

The Wake Forest School of Medicine Animal Care and Use Committee approved all protocols and procedures performed in this study. This study does not include any human subjects.

## Supporting information


**Figure S1.** Effect of intratracheal lipopolysaccharide‐induced acute lung injury. (a) Total cell count in BAL in mice with no ALI (day 0) and at days 2, 3, 7, 10, 25–29. (b) Representative flow cytometry analysis gating for CD45^+^CD11b^+^, neutrophils, macrophages, monocytes, and dendritic cells. (c) Representative gating for CD163 and CD206 expression of M2‐like polarization (CD206^+^CD163^+^) and MHCII expression of M1‐like polarization (MHCII^+^CD163^−^ and MHCII^+^CD206^−^). The gates for CD11c, F4/80, MHC II, CD163, and CD206 were determined by comparing cells stained with the antibodies to fluorescence minus one (FMO) control cells in which the antibody was omitted from the antibody cocktail. D: Representative FMO for F4/80.
**Figure S2:** (a) Mean fluorescent intensity of F4/80 expression in total CD45^+^CD11b^+^ leukocytes from skeletal muscle treated with intramuscular clodronate and vehicle control. (b) Representative F4/80 fluorescent intensity in representative mouse muscle treated with intramuscular clodronate versus vehicle control in the contralateral leg. (c) Systemic clodronate effect on total cell count in BAL. (d) Systemic clodronate effect on percent of monocytes/macrophages in the BAL. Values are expressed as mean ± SD. A nonparametric *t*‐test (Mann–Whitney test) was performed.
**Figure S3:** Muscle MuRF1 transcription following intramuscular and systemic clodronate treatment. Total RNA was isolated from muscles from the intramuscular and systemic clodronate experiments using the Trizol reagent per the manufacturer’s instructions (Invitrogen). The RNA was reverse transcribed into cDNA and amplified with the appropriate primers using a one‐step kit (Lo‐Rox Bio‐78,005 Bioline) and a Thermo cycler (7500 Fast real time PCR system, Applied Biosystems). All mRNA expression was normalized to GAPDH. TaqMan probe‐based primers (Applied Biosystems) were used in all reactions. Accession numbers are listed after each gene: GAPDH, Mm99999915_g1; Trim63 (MuRF1), Mm01185221_m1.A. A: MuRF1 fold change in intramuscular clodronate treatment versus vehicle. B: MuRF1 fold change in systemic clodronate treatment versus vehicle. Values are expressed as mean ± SD. Wilcoxon signed‐rank test (paired nonparametric *t* Test) was performed (a). A nonparametric t‐test (Mann–Whitney test) was performed (b).

## Data Availability

The data that support the findings of this study are available from the corresponding author upon reasonable request.
