# Supplementary figures and images for: Macrophages modulate skeletal muscle wasting and recovery in acute lung injury in mice
Source: Physiol Rep. 2024 Sep 26;12(18):e70052. doi: 10.14814/phy2.70052 (PMC11427096; doi:10.14814/phy2.70052)

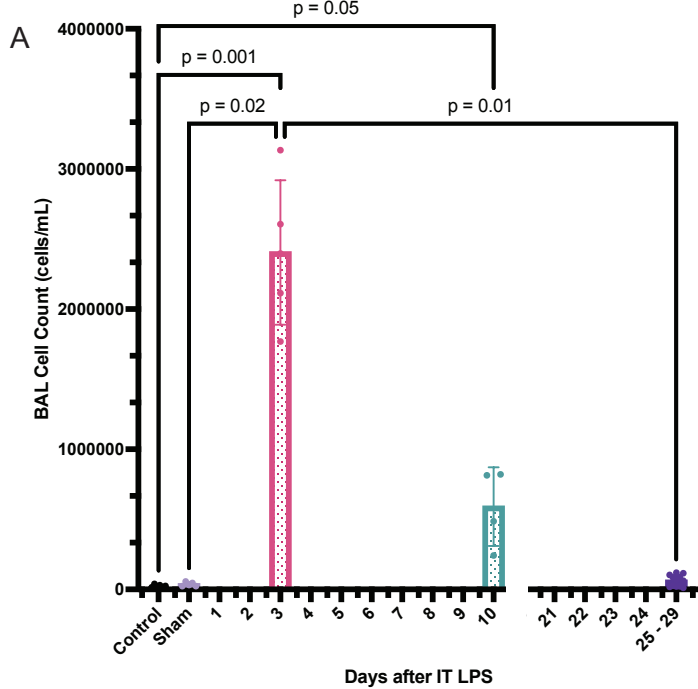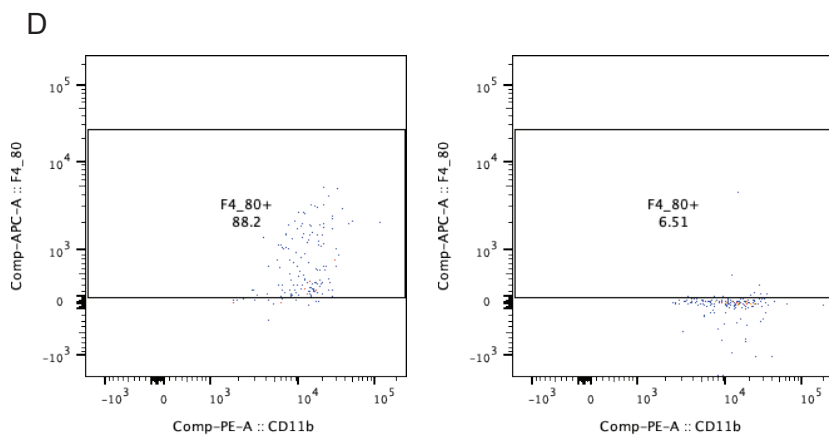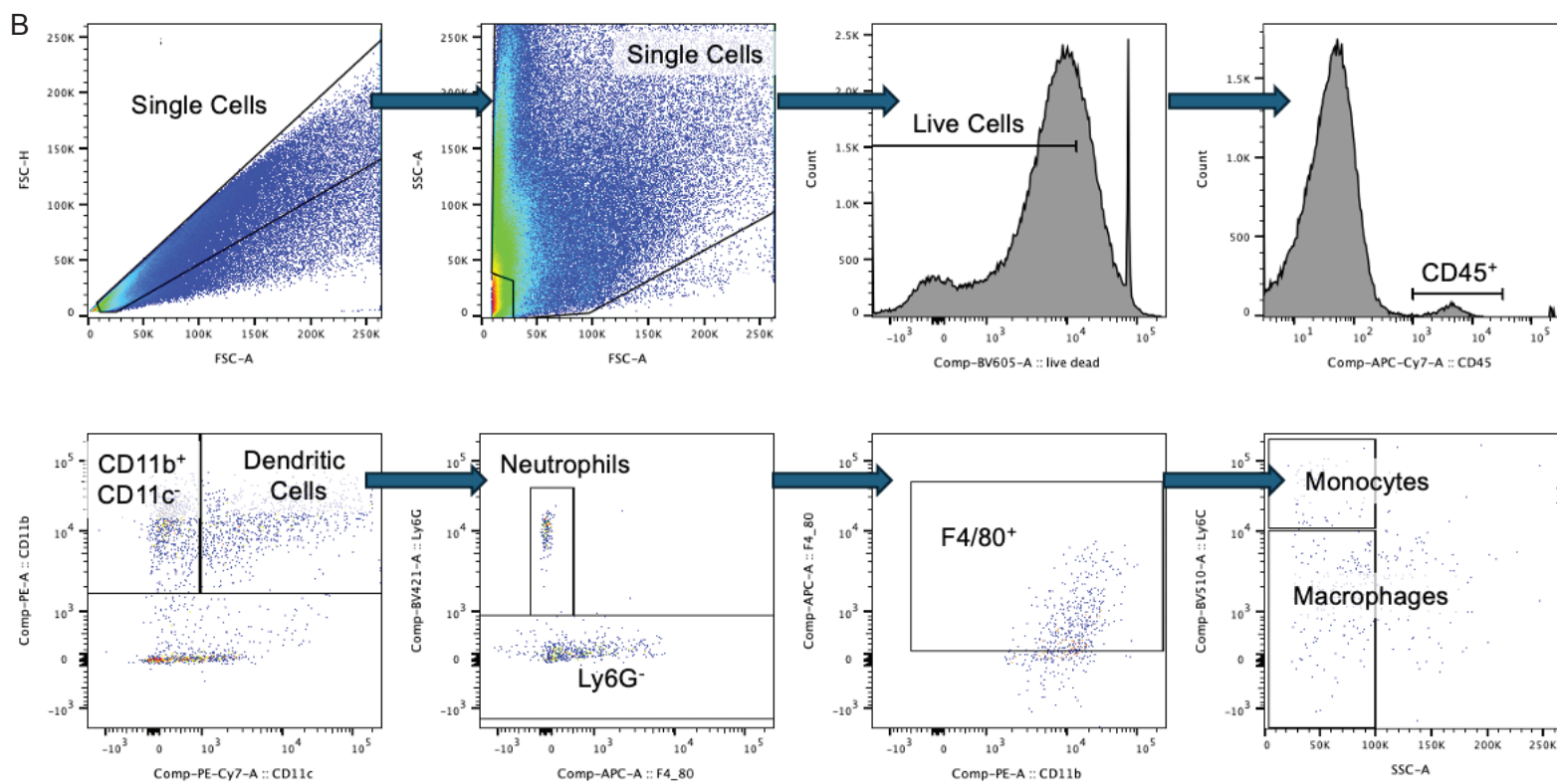

Anti-inflammatory

Pro-inflammatory

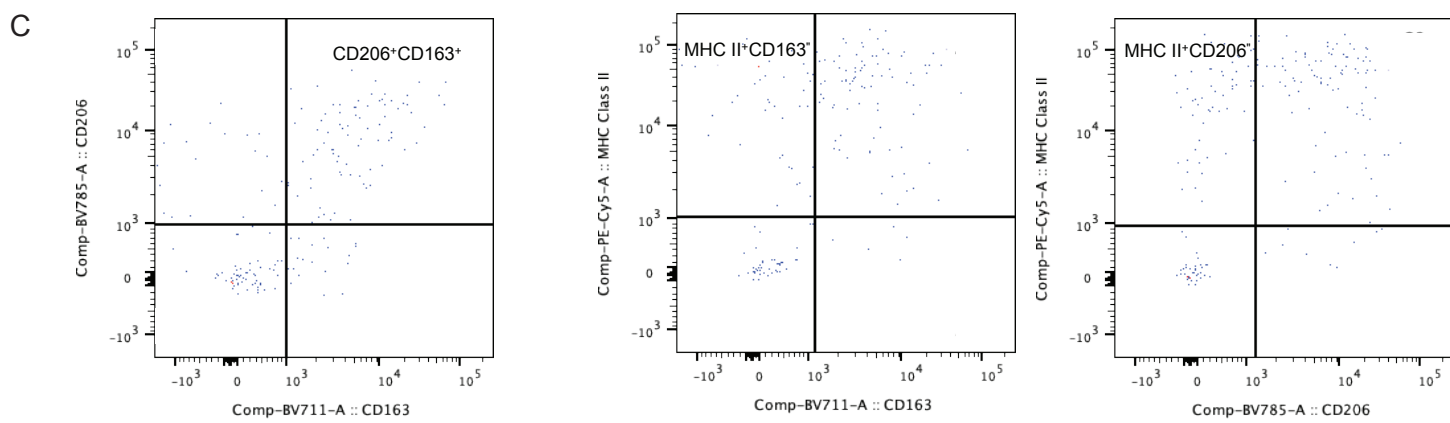

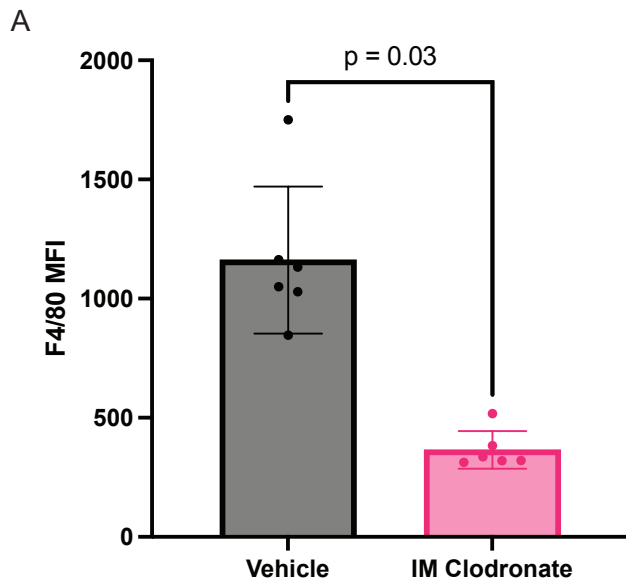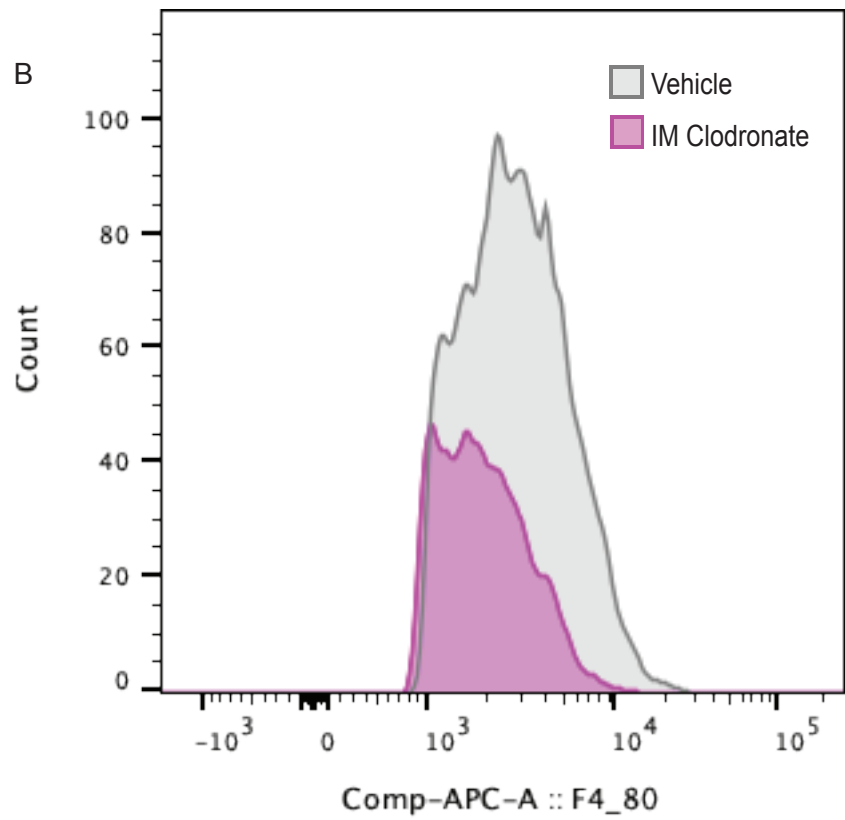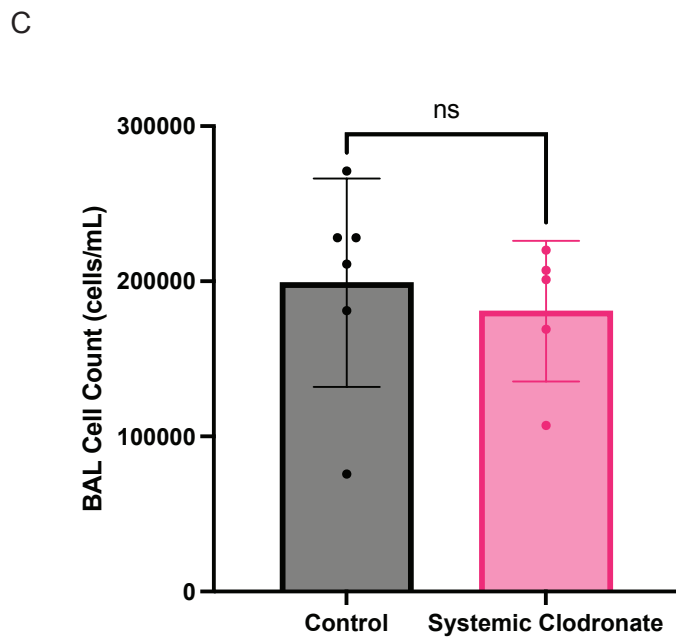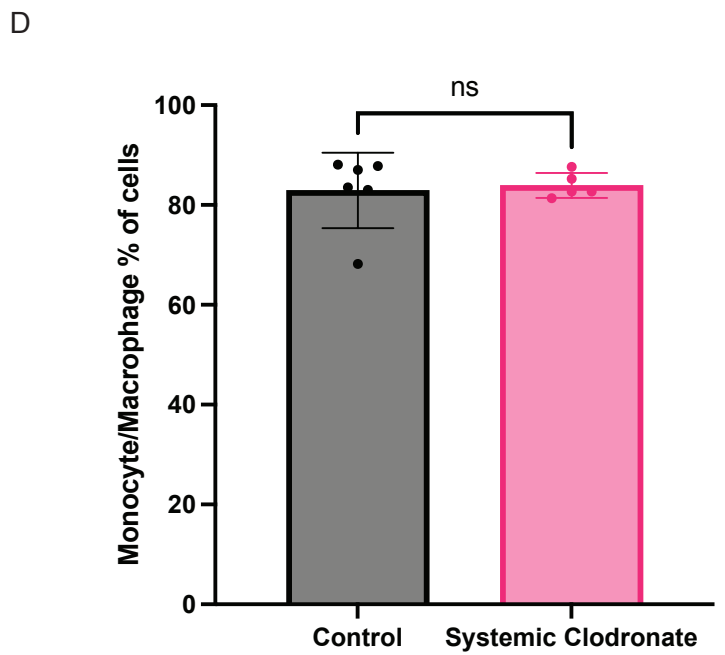

A

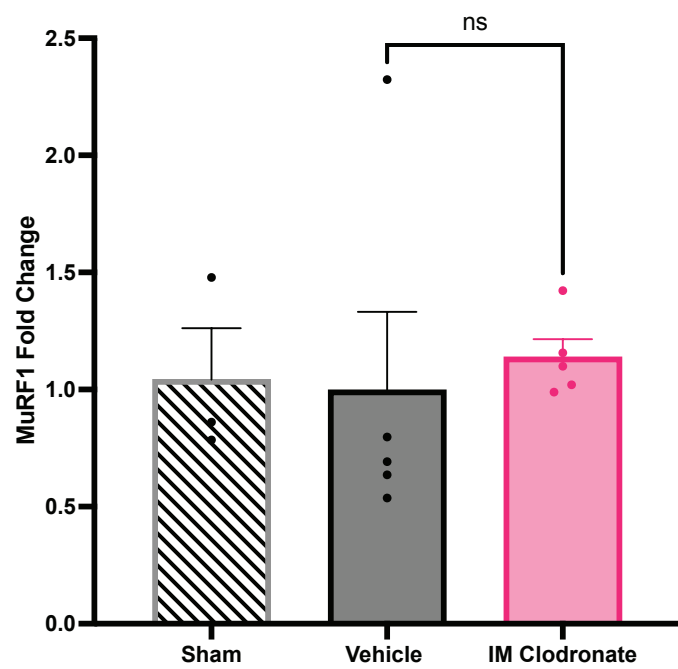

B

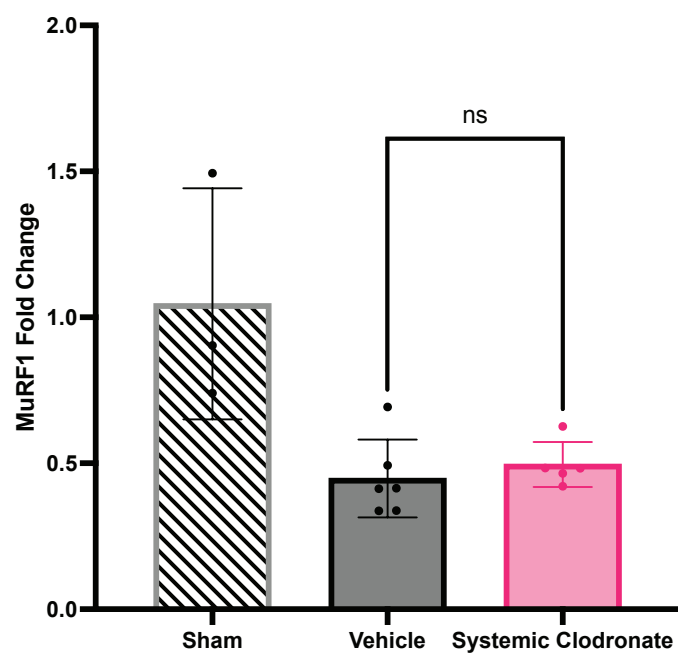

Supplement: Supplementary file 1 — Figure S1. Effect of intratracheal lipopolysaccharide‐induced acute lung injury. (a) Total cell count in BAL in mice with no ALI (day 0) and at days 2, 3, 7, 10, 25–29. (b) Representative flow cytometry analysis gating for CD45+CD11b+, neutrophils, macrophages, monocytes, and dendritic cells. (c) Representative gating for CD163 and CD206 expression of M2‐like polarization (CD206+CD163+) and MHCII expression of M1‐like polarization (MHCII+CD163− and MHCII+CD206−). The gates for CD11c, F4/80, MHC II, CD163, and CD206 were determined by comparing cells stained with the antibodies to fluorescence minus one (FMO) control cells in which the antibody was omitted from the antibody cocktail. D: Representative FMO for F4/80. Figure S2: (a) Mean fluorescent intensity of F4/80 expression in total CD45+CD11b+ leukocytes from skeletal muscle treated with intramuscular clodronate and vehicle control. (b) Representative F4/80 fluorescent intensity in representative mouse muscle treated with intramuscular clodronate versus vehicle control in the contralateral leg. (c) Systemic clodronate effect on total cell count in BAL. (d) Systemic clodronate effect on percent of monocytes/macrophages in the BAL. Values are expressed as mean ± SD. A nonparametric t‐test (Mann–Whitney test) was performed. Figure S3: Muscle MuRF1 transcription following intramuscular and systemic clodronate treatment. Total RNA was isolated from muscles from the intramuscular and systemic clodronate experiments using the Trizol reagent per the manufacturer’s instructions (Invitrogen). The RNA was reverse transcribed into cDNA and amplified with the appropriate primers using a one‐step kit (Lo‐Rox Bio‐78,005 Bioline) and a Thermo cycler (7500 Fast real time PCR system, Applied Biosystems). All mRNA expression was normalized to GAPDH. TaqMan probe‐based primers (Applied Biosystems) were used in all reactions. Accession numbers are listed after each gene: GAPDH, Mm99999915_g1; Trim63 (MuRF1), Mm01185221_m1.A. A: MuRF1 [file PHY2-12-e70052-s001.pdf]
